# A *TREML2* missense variant influences specific hippocampal subfield volumes in cognitively normal elderly subjects

**DOI:** 10.1002/brb3.1573

**Published:** 2020-02-19

**Authors:** Si‐Yu Wang, Xiao Xue, Rui Duan, Peng‐Yu Gong, Yan E, Teng Jiang, Ying‐Dong Zhang, For the Alzheimer’s Disease Neuroimaging Initiative

**Affiliations:** ^1^ Department of Neurology Nanjing First Hospital Nanjing Medical University Nanjing China; ^2^ School of Basic Medicine & Clinical Pharmacy China Pharmaceutical University Nanjing China

**Keywords:** ADNI, Alzheimer' s disease, CA1, *TREML2*

## Abstract

**Introduction:**

Triggering receptor expressed on myeloid cells‐like transcript 2 gene (*TREML2*) is a newly identified AD susceptibility gene. Its missense variant rs3747742‐C substantially decreases AD risk in both Caucasians and Han Chinese, but the underlying mechanisms remain elusive. In the present study, to uncover the possible mechanisms by which *TREML2* rs3747742‐C reduces AD risk, we investigated the possible relation of this variant with AD‐related brain structures using a cognitively normal elderly population from Alzheimer's Disease Neuroimaging Initiative (ADNI) database.

**Methods:**

In total, 158 cognitively normal elders from ADNI database with complete data for brain structures and *TREML2* rs3747742 genotype were included in this study. The association of *TREML2* rs3747742 genotype with the structures of three cerebral cortices (entorhinal cortex, middle temporal gyrus, and parahippocampal gyrus), two subcortical regions (amygdala and hippocampus), and three subfields of hippocampus (CA1, CA2 + CA3, and CA4 + dentate gyrus) was investigated.

**Results:**

A significant difference was noted in the volume of right CA1 subfield among three genotypes of *TREML2* rs3747742 (*p* = .0364). In the multivariate analysis, *TREML2* rs3747742‐C significantly increased right CA1 subfield volume after adjusting for age, gender, education years, *APOE* ε4 status, and intracranial volume under the recessive genetic model (Bonferroni corrected *p* = .003586).

**Conclusion:**

The present study provides the first evidence that *TREML2* rs3747742‐C carriers have larger volumes of hippocampal CA1 subfield in a cognitively normal elderly population. These findings imply that enhancement of brain reserve may contribute to the protection of *TREML2* rs3747742‐C in AD susceptibility.

## Graphical abstract

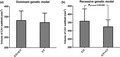

## 1 INTRODUCTION

Alzheimer's disease (AD) is the most common form of dementia in the world, affecting 5% of the population over 65 years (Alzheimer's, 2016; Lane, Hardy, & Schott, 2018). The late‐onset AD accounts for the majority of all AD cases and is considered as a genetically complex disorder that caused by an interaction between environmental factors and susceptibility genes (Jiang, Yu, Tian, & Tan, 2013). Investigating the mechanisms by which variants of these susceptibility genes modify AD risk will provide valuable insights into the pathogenesis of this devastating disease.

Triggering receptor expressed on myeloid cells*‐*like transcript 2 gene (*TREML2*) is a newly identified AD susceptibility gene. Its missense variant rs3747742‐C is revealed to substantially reduce AD risk in both Caucasians and Han Chinese (Benitez et al., 2014; Jiang et al., 2017), but the underlying mechanisms are still elusive. Previous studies investigated the association of *TREML2* rs3747742 with cerebrospinal fluid (CSF) biomarkers of AD and found that carriers of C allele had a decreased level of both hyperphosphorylated and total CSF tau (Benitez et al., 2013; Song et al., 2019), indicating that *TREML2* rs3747742‐C may decrease AD risk by attenuating neurodegeneration process.

In the present study, to uncover the other possible mechanisms by which *TREML2* rs3747742‐C reduces AD risk, we investigated the possible relation of this variant with AD‐related brain structures using a cognitively normal elderly population from Alzheimer's Disease Neuroimaging Initiative (ADNI) database. For the first time, we showed that *TREML2* rs3747742‐C carriers have larger volumes of hippocampal CA1 subfield after adjusting for age, gender, education years, *APOE* ε4 status, and intracranial volume. These findings imply that enhancement of brain reserve may also contribute to the protection of *TREML2* rs3747742‐C in AD susceptibility.

## 2 METHODS

### 2.1 About ADNI database and subject selection

All data used in this article were obtained from the ADNI database (http://adni.loni.usc.edu). The ADNI was launched in 2003 as a public‐private partnership, led by Principal Investigator Michael W. Weiner, MD. The primary goal of ADNI has been to test whether serial magnetic resonance imaging (MRI), positron emission tomography (PET), other biological markers, and clinical and neuropsychological assessment can be combined to measure the progression of mild cognitive impairment (MCI) and early Alzheimer's disease (AD). For up‐to‐date information, see http://www.adni-info.org. In total, 158 cognitively normal elders from ADNI database with complete data for brain structures and *TREML2* rs3747742 genotype were included in this study.

### 2.2 Ethical approval

As per ADNI protocols, all procedures performed in studies involving human participants were in accordance with the 1964 Helsinki declaration and its later amendments or comparable ethical standards. More details can be found at adni.loni.usc.edu.

### 2.3 MRI for brain structures

MRI data of brain structures were extracted from ADNI website (*TREML2* rs3747742 C/C genotype: 12 subjects, *TREML2* rs3747742 C/T genotype: 71 subjects, *TREML2* rs3747742 T/T genotype: 75 subjects). The details regarding image acquisition and process can be found at http://adni.loni.usc.edu/methods. In this study, three cerebral cortices (entorhinal cortex, middle temporal gyrus, and parahippocampal gyrus) and two subcortical structures (amygdala and hippocampus) that associated with AD were defined as ROIs. Regarding the subfields of hippocampus, three regions (CA1, CA2 + CA3, and CA4 + dentate gyrus) were selected as ROIs.

### 2.4 Statistical analysis

Statistical analyses were carried out by GraphPad Prism 6 (GraphPad Software), R 3.12 (http://www.r-project.org/) and PLINK 1.07 (http://pngu.mgh.harvard.edu/wpurcell/plink/). In univariate analysis, the association of *TREML2* rs3747742 genotypes with AD‐related brain structures was investigated using one‐way ANOVA. In multivariate analysis, the association between *TREML2* rs3747742 genotypes and AD‐related brain structures was analyzed using a multiple linear regression adjusting for age, gender, education years, *APOE* ε4 status, and intracranial volume under dominant and recessive genetic models. These two genetic models were defined as follows: (C/C + C/T) versus T/T for the dominant model, C/C versus (C/T + T/T) for the recessive model. Bonferroni correction was used for the adjustment of multiple comparisons. *p* < .05 was considered significant.

## 3 RESULTS

As indicated by Table S1 and Table S2, no difference was observed in the average thickness and volume of AD‐related cerebral cortices including bilateral entorhinal cortices, middle temporal gyri, and parahippocampal gyri among three genotypes of *TREML2* rs3747742. Meanwhile, no difference was noted in the volume of subcortical structures including bilateral amygdalae and hippocampi among three genotypes of *TREML2* rs3747742 (Table S3).

**Figure 1:**
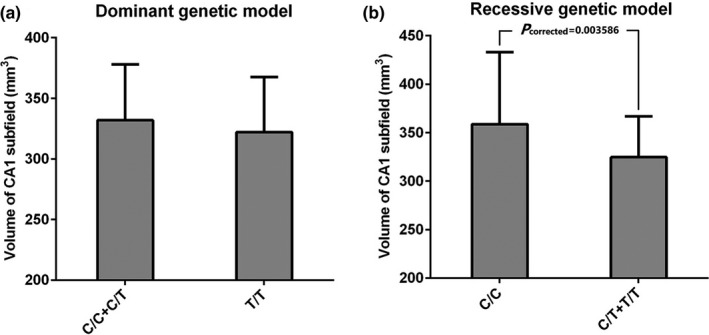
The influence of *TREML2* rs3747742‐C on right CA1 subfield volume. (a) The association between *TREML2* rs3747742‐C and right CA1 subfield volume after adjusting for age, gender, education years, *APOE* ε4 status, and intracranial volume under the dominant genetic model ([C/C + C/T] vs. T/T). (b) The association between *TREML2* rs3747742‐C and right CA1 subfield volume after adjusting for age, gender, education years, *APOE* ε4 status, and intracranial volume under the recessive genetic model (C/C vs. [C/T + T/T])

Regarding the hippocampal subfields, a significant difference was found in the volume of right CA1 subfield among three genotypes of *TREML2* rs3747742 (*p* = .0364, see Table 1). We then investigated the association between *TREML2* rs3747742 and right CA1 subfield volume using multiple linear regression adjusting for age, gender, education years, *APOE* ε4 status, and intracranial volume under dominant and recessive genetic models. As revealed by Figure 1, *TREML2* rs3747742‐C significantly increased right CA1 subfield volume under the recessive genetic model after Bonferroni correction (*P*c = 0.003586).

**Table 1:** The influence of *TREML2* rs3747742 genotypes on the volume of hippocampal subfields

Hippocampal subfields	Genotypes			*p* value
	C/C	C/T	T/T	
CA1 subfield (Left, mm^3^)	334.5 ± 62.15 (12)	316.5 ± 42.16 (71)	321.1 ± 43.77 (75)	.4191
CA1 subfield (Right, mm^3^)	358.7 ± 74.23 (12)	327.4 ± 38.47 (71)	322.1 ± 45.48 (75)	.0364
CA2 + CA3 subfields (Left, mm^3^)	909.4 ± 103.8 (12)	911.5 ± 137.2 (71)	903.9 ± 129.1 (75)	.9397
CA2 + CA3 subfields (Right, mm^3^)	981.3 ± 111.1 (12)	968.5 ± 135.5 (71)	947.4 ± 150.4 (75)	.5713
CA4 + dentate gyrus subfields (Left, mm^3^)	506.9 ± 56.2 (12)	512.8 ± 71.32 (71)	510.2 ± 73.27 (75)	.9541
CA4 + dentate gyrus subfields (Right, mm^3^)	535.9 ± 59.21 (12)	539.2 ± 74.61 (71)	524.9 ± 80.2 (75)	.5195

Note

Data in this table are expressed as mean ± *SD* (number of subjects) and analyzed with one‐way ANOVA.

## 4 DISCUSSION

As a newly identified susceptibility gene for AD, *TREML2* locates on chromosome 6p21.1‐q15, a region showing a strong association with AD risk (Lambert et al., 2013). Previously, by mining Caucasian exome‐sequencing datasets, Benitez and colleagues revealed that *TREML2* rs3747742‐C was associated with a lowered risk of AD (Benitez et al., 2014). Recently, we had confirmed this association in a large Han Chinese population including 992 AD patients and 1,358 healthy controls (Jiang et al., 2017), further supporting a protective role of *TREML2* rs3747742‐C in AD susceptibility.

However, the underlying mechanisms by which *TREML2* rs3747742‐C reduces AD risk remain largely unknown. Benitez et al. recently found that *TREML2* rs3747742‐C carriers had a decreased level of CSF hyperphosphorylated tau (Benitez et al., 2013). Meanwhile, Song and colleagues revealed that *TREML2* rs3747742‐C was related to a reduced CSF total tau level in AD patients after controlling for age, gender, education, and *APOE* ε4 status (Song et al., 2019). Since CSF tau levels reflect the degree of neurodegeneration (Frost, Gotz, & Feany, 2015), these findings implied that *TREML2* rs3747742‐C might modify AD risk by attenuating neurodegeneration process. In the present study, we investigated the possible relation of *TREML2* rs3747742‐C with AD‐related brain structures using a cognitively normal elderly population from ADNI database. For the first time, we revealed that *TREML2* rs3747742‐C carriers had larger volumes of hippocampal CA1 subfield after adjusting for age, gender, education years, *APOE* ε4 status, and intracranial volume. The hippocampal CA1 subfield, also known as Sommer's sector, is required for the retrieval of spatial and contextual memory (Guerreiro et al., 2013; Thomas, 2015). Since CA1 subfield is particularly susceptible to cytotoxicity (Yang, Tian, Yang, & Zhang, 2010), a larger volume of this region might provide a better compensation for neuropathological damages during AD progression. Therefore, it seemed that enhancement of brain reserve might also contribute to the protection of *TREML2* rs3747742‐C in AD susceptibility.

Triggering receptor expressed on myeloid cells‐like transcript 2 gene encodes a single‐pass type I membrane protein that belongs to the immunoglobulin superfamily (Klesney‐Tait, Turnbull, & Colonna, 2006). In the brain, TREML2 is mainly expressed by microglia, the immune cell within the central nervous system (Zheng et al., 2016). In a previous functional study, Zheng and colleagues showed that the expression of TREML2 on microglia was upregulated by oligomeric amyloid‐β stimulation (Zheng et al., 2016). More importantly, knockdown of TREML2 facilitated microglia proliferation and suppressed microglia‐mediated release of proinflammatory cytokines (Zheng et al., 2016). These findings implied that TREML2 might be a modulator of microglia during AD progression. As a missense variant, *TREML2* rs3747742‐C leads to a change of amino acids at 144 residue (p.S144G) of TREML2, and functional studies are warranted to determine whether the protective effects of *TREML2* rs3747742‐C against AD susceptibility are related to the alteration of TREML2 protein and microglia functions. However, according to the prediction of MutationTaster and PolyPhen‐2 software (Adzhubei, Jordan, & Sunyaev, 2013; Adzhubei et al., 2010; Schwarz, Cooper, Schuelke, & Seelow, 2014), p.S144G amino acid change was unlikely to affect the structure or functions of TREML2 protein. Therefore, it is also possible that the *TREML2* rs3747742‐C is in LD with other nearby functional variants and thus protects against AD risk (Carrasquillo et al., 2017).

The main limitation of this study is the relatively small sample size of the cognitively normal elders in the ADNI dataset. Therefore, our findings should be further validated using a larger cohort in the future.

In conclusion, the present study provides the first evidence that *TREML2* rs3747742‐C carriers have larger volumes of hippocampal CA1 subfield after adjusting for age, gender, education years, *APOE* ε4 status, and intracranial volume in a cognitively normal elderly population. These findings imply that enhancement of brain reserve may also contribute to the protection of *TREML2* rs3747742‐C in AD susceptibility.

## CONFLICT OF INTEREST

The authors confirm that this article has no conflict of interest.

## AUTHORS' CONTRIBUTION

Si‐Yu Wang and Xiao Xue were involved in data analysis. Rui Duan and Peng‐Yu Gong were involved in data acquisition. Yan E and Ying‐Dong Zhang were involved in data interpretation. Teng Jiang was involved in manuscript preparation. The investigators within the ADNI contributed to the design and implementation of ADNI database.

## INFORMED CONSENT

A written informed consent was obtained from each participant or the legal guardian. More details can be found at adni.loni.usc.edu.

## Data Availability

The data that support the findings of this study are available from the corresponding author upon reasonable request.
