# Supplementary material for: A TREML2 missense variant influences specific hippocampal subfield volumes in cognitively normal elderly subjects
Source: Brain Behav. 2020 Feb 19;10(4):e01573. doi: 10.1002/brb3.1573 (PMC7177563; doi:10.1002/brb3.1573)
Supplement: Supplementary file 1 [file BRB3-10-e01573-s001.docx]

**Supplementary Table S1. The influence of *TREML2* rs3747742 genotypes on the** **average thickness of AD-related cerebral cortices.**

| Cerebral cortices | Genotypes | | | *P* value |
| --- | --- | --- | --- | --- |
|  | C/C | C/T | T/T |  |
| Entorhinal cortex (Left, mm) | 3.465±0.3043 (12) | 3.454±0.3122 (71) | 3.513±0.3171 (75) | 0.5158 |
| Entorhinal cortex (Right, mm) | 3.61±0.4139 (12) | 3.556±0.3705 (71) | 3.653±0.3523 (75) | 0.2791 |
| Middle temporal gyrus (Left, mm) | 2.712±0.1416 (12) | 2.77±0.1712 (71) | 2.786±0.1508 (75) | 0.3238 |
| Middle temporal gyrus (Right, mm) | 2.793±0.0719 (12) | 2.82±0.1423 (71) | 2.843±0.1442 (75) | 0.3983 |
| Parahippocampal gyrus (Left, mm) | 2.675±0.2891 (12) | 2.757±0.3558 (71) | 2.844±0.3362 (75) | 0.1473 |
| Parahippocampal gyrus (Right, mm) | 2.705±0.2263 (12) | 2.722±0.279 (71) | 2.769±0.2448 (75) | 0.4802 |

Data in this table are expressed as mean±SD (number of subjects) and analyzed with one-way ANOVA.

**Supplementary Table S2. The influence of *TREML2* rs3747742 genotypes on the volume of AD-related cerebral cortices.**

| Cerebral cortices | Genotypes | | | *P* value |
| --- | --- | --- | --- | --- |
|  | C/C | C/T | T/T |  |
| Entorhinal cortex (Left, mm^3^) | 1976±314.6 (12) | 1961±340.4 (71) | 1988±301 (75) | 0.8785 |
| Entorhinal cortex (Right, mm^3^) | 1766±316.5 (12) | 1878±407.2 (71) | 1891±422.9 (75) | 0.6167 |
| Middle temporal gyrus (Left, mm^3^) | 9450±1202 (12) | 9895±1329 (71) | 9505±1196 (75) | 0.1441 |
| Middle temporal gyrus (Right, mm^3^) | 10960±1448 (12) | 10962±1357 (71) | 10924±1300 (75) | 0.9844 |
| Parahippocampal gyrus (Left, mm^3^) | 2128±388.9 (12) | 2081±334.6 (71) | 2144±311.4 (75) | 0.5062 |
| Parahippocampal gyrus (Right, mm^3^) | 1984±267.5 (12) | 1972±287.4 (71) | 2020±294.8 (75) | 0.6006 |

Data in this table are expressed as mean±SD (number of subjects) and analyzed with one-way ANOVA.

**Supplementary Table S3. The influence of *TREML2* rs3747742 genotypes on the volume of AD-related** **subcortical structures.**

| Subcortical structures | Genotypes | | | *P* value |
| --- | --- | --- | --- | --- |
|  | C/C | C/T | T/T |  |
| Amygdala (Left, mm^3^) | 1465±204.1 (12) | 1397±241.1 (71) | 1391±184.4 (75) | 0.5335 |
| Amygdala (Right, mm^3^) | 1508±210.9 (12) | 1447±237.3 (71) | 1447±181.3 (75) | 0.6284 |
| Hippocampus (Left, mm^3^) | 3600±441 (12) | 3660±461 (71) | 3643±447.4 (75) | 0.9079 |
| Hippocampus (Right, mm^3^) | 3659±416.5 (12) | 3723±480.8 (71) | 3700±488.7 (75) | 0.8984 |

Data in this table are expressed as mean±SD (number of subjects) and analyzed with one-way ANOVA.
